# Consonant and Vowel Confusions in Well-Performing Children and Adolescents With Cochlear Implants, Measured by a Nonsense Syllable Repetition Test

**DOI:** 10.3389/fpsyg.2019.01813

**Published:** 2019-08-14

**Authors:** Arne Kirkhorn Rødvik, Ole Tvete, Janne von Koss Torkildsen, Ona Bø Wie, Ingebjørg Skaug, Juha Tapio Silvola

**Affiliations:** ^1^Department of Special Needs Education, Institute of Educational Sciences, University of Oslo, Oslo, Norway; ^2^Cochlear Implant Unit, Department of Otorhinolaryngology, Division of Surgery and Clinical Neuroscience, Oslo University Hospital, Oslo, Norway; ^3^Cochletten Foundation, Oslo, Norway; ^4^Ear, Nose, and Throat Department, Division of Surgery, Akershus University Hospital, Lørenskog, Norway

**Keywords:** cochlear implants, speech perception, speech sound confusions, consonants, vowels, hearing

## Abstract

Although the majority of early implanted, profoundly deaf children with cochlear implants (CIs), will develop correct pronunciation if they receive adequate oral language stimulation, many of them have difficulties with perceiving minute details of speech. The main aim of this study is to measure the confusion of consonants and vowels in well-performing children and adolescents with CIs. The study also aims to investigate how age at onset of severe to profound deafness influences perception. The participants are 36 children and adolescents with CIs (18 girls), with a mean (*SD*) age of 11.6 (3.0) years (range: 5.9–16.0 years). Twenty-nine of them are prelingually deaf and seven are postlingually deaf. Two reference groups of normal-hearing (NH) 6- and 13-year-olds are included. Consonant and vowel perception is measured by repetition of 16 bisyllabic vowel-consonant-vowel nonsense words and nine monosyllabic consonant-vowel-consonant nonsense words in an open-set design. For the participants with CIs, consonants were mostly confused with consonants with the same voicing and manner, and the mean (*SD*) voiced consonant repetition score, 63.9 (10.6)%, was considerably lower than the mean (*SD*) unvoiced consonant score, 76.9 (9.3)%. There was a devoicing bias for the stops; unvoiced stops were confused with other unvoiced stops and not with voiced stops, and voiced stops were confused with both unvoiced stops and other voiced stops. The mean (*SD*) vowel repetition score was 85.2 (10.6)% and there was a bias in the confusions of [i:] and [y:]; [y:] was perceived as [i:] twice as often as [y:] was repeated correctly. Subgroup analyses showed no statistically significant differences between the consonant scores for pre- and postlingually deaf participants. For the NH participants, the consonant repetition scores were substantially higher and the difference between voiced and unvoiced consonant repetition scores considerably lower than for the participants with CIs. The participants with CIs obtained scores close to ceiling on vowels and real-word monosyllables, but their perception was substantially lower for voiced consonants. This may partly be related to limitations in the CI technology for the transmission of low-frequency sounds, such as insertion depth of the electrode and ability to convey temporal information.

## Introduction

Provided with adequate access to environments in which speech is the common mode of communication, the majority of profoundly deaf children implanted in their sensitive period (before age 3.5–4.0 years) will develop intelligible speech and functional hearing for oral language (Kral and Sharma, 2012; Leigh et al., 2013; Dettman et al., 2016). Early implanted children follow similar development in speech and language as normal-hearing (NH) children do (e.g., the systematic review by Bruijnzeel et al., 2016). However, early implanted children with good speech perception ability do not discriminate minute details of speech, such as voicing, frication, and nasality, as well as their NH peers, even in quiet surroundings (Tye-Murray et al., 1995; Geers et al., 2003).

The present study aims to reveal possible systematic misperceptions of speech sounds in detail for children and adolescents with cochlear implants (CIs) and to investigate how age at onset of severe to profound (pre-, peri-, and postlingual) deafness influences their confusion of speech sounds and features. In the following, we will outline the maturation of the auditory system and the fundamentals of speech processing in CIs, before presenting the rationale for our test design and giving a brief introduction to the Norwegian language.

The human cochlea is fully developed at birth, but the brain’s auditory pathways and centers, from the brain stem to the auditory cortex, continue to develop. Conditions for the acquisition of language are optimal in a sensitive period, which can be estimated by measuring the cortical P1 latency response as an index of maturation of the auditory pathway in populations with abnormal auditory experience, such as congenital profound deafness. Sharma et al. (2002a, b, c) found that the optimal sensitive period for cochlear implantation in profoundly deaf children lasts until approximately 3.5–4 years of age, and it is important that children receive auditory stimulation within this critical period. These children can still benefit from CIs until the eventual end of the overall sensitive period, at approximately 6.5–7.0 years of age (Kral and Sharma, 2012). However, later implantation in congenitally deaf children normally results in difficulties with acquiring oral speech and language skills.

As normal maturation of the auditory system depends on adequate auditory input in very early childhood, detection of hearing loss by otoacoustic emissions and/or auditory brainstem responses right after birth is crucial. Immediate programming of hearing aids (HAs) for infants with discovered mild to moderate hearing loss, or of CIs for the profoundly deaf among them, will facilitate stimulation of the brain’s auditory pathways in the sensitive period. Clinical findings indisputably show that children with hearing impairments who receive appropriate and

early intervention achieve much better hearing and better oral language performance than those who start the process later (Wilson and Dorman, 2008; Niparko et al., 2010; Wie, 2010).

The gradual development and maturation of the auditory system can be seen in outcomes of auditory tests into the late teenage years, with individual variability within a given age (Maxon and Hochberg, 1982; Fischer and Hartnegg, 2004). Children’s peripheral hearing is established before their speech. However, the development of the ability to discriminate speech sounds, as well as vocabulary and language, takes many years.

Auditory sensitivity in audiometric tests, in absence of noise or other masking stimuli, is known to improve between infancy and early school age (Olsho et al., 1988; Trehub et al., 1988). Litovsky (2015) suggests that the reason for this improvement is that the tasks used to measure perception of pure-tones do not separate the effects of cognitive ability, motivation, memory, and variability in neural representation of the stimuli. For real-word tests, top-down processing allows for decoding based on context and is facilitated by the lexical content present in real-word stimulus materials or by the intrinsic language proficiency. To diminish the influence of these factors in the present study, auditory skills are measured by a nonsense syllable repetition test (NSRT), which is idealized to measure the perception of speech sounds with only minor influence from top-down processing and with minimal stress on working memory. This test should therefore establish a more correct expression of the true auditory perception skills of a child with CIs.

CI users are often classified into pre-, peri-, and postlingually deaf. In the present study, prelingual deafness is defined as congenital, profound deafness or onset of severe to profound deafness before the age of 12 months. According to the widely used definition by the World Health Organization [WHO] (2019), severe hearing loss is characterized by a pure-tone average (PTA)^1^ between a 60 and 80 dB hearing level (HL), and profound hearing loss is characterized by a PTA above 80 dB HL. In prelingually deaf children, the auditory system is immature when hearing is initiated by a CI, whose stimulus signal is different from the signal generated by the inner hair cells in a normal cochlea. The earlier the age at implantation, the faster the adaptation to the novel signal, and the better the speech perception outcomes (Niparko et al., 2010; Tobey et al., 2013; Liu et al., 2015). Furthermore, prelingually deaf children with CIs can be divided into two groups: those who have had no or minimal access to sound and hence acquired very little oral language before implantation (these children are often congenitally deaf and receive a CI before age 1), and those who have acquired oral language and benefited from HAs due to residual hearing, receiving a CI at a higher age.

The children with onset of severe to profound deafness between 1 and 3 years of age are classified as perilingually deaf. postlingual deafness is defined as progressive or sudden hearing loss and onset of severe to profound deafness after age 3 years, with a benefit from HAs and acquired oral language before onset of deafness (Myhrum et al., 2017).

Although language acquisition is a gradual process, the breakpoint of age 1 year for distinguishing between pre- and perilingual deafness is precisely defined for practical reasons. This age corresponds to when infants usually start saying their first words (Darley and Winitz, 1961; Locke, 1983, p. 8). In postlingually deaf adults and children, the neural pathways in the brain have been shaped by acoustic sound perception before onset of deafness. The degree of success with a CI is dependent on how the brain compares the new signal with what was heard previously.

For both the pre-, peri-, and postlingually deaf, auditory deprivation will occur after a period of lack of sensory input. This process entails a degeneration of the auditory system, both peripherally and centrally (Feng et al., 2018), including a degradation of neural spiral ganglion cells (Leake and Hradek, 1988). If profound deafness occurs in the sensitive period before 3.5–4.0 years of age, it arrests the normal tonotopic organization of the primary auditory cortex. This arrest can, however, be reversed after reactivation of afferent input by a CI (Kral, 2013).

The hearing-impaired participants in this study are aided by CIs, which consist of a speech processor on the ear and a surgically implanted electrode array in the cochlea with up to 22 electrical contacts. A speech signal input is received by the built-in speech processor microphone and translated into sequences of electrical pulses in the implant by a stimulation strategy. The main purpose of every such strategy is to set up an electrical signal in the auditory nerve using electrical stimulation patterns in the electrode array to mimic the signal in a normal ear. These patterns vary somewhat between stimulation strategies and implant manufacturers, but they all attempt to convey spectral (frequency-related) and temporal information of the original signal through the implant (Wouters et al., 2015).

The spectral information of the speech signal (e.g., the first and second formant, F1 and F2) is conveyed by the multichannel organization of the implants, by mimicking the tonotopic (place) organization of the cochlea from low frequencies in the apex to high frequencies in the base. This information is implemented in all stimulation strategies from the main (in terms of market share) implant manufacturers today, listed in alphabetical order: Advanced Bionics (Stäfa, Switzerland), Cochlear (Sydney, NSW, Australia), Med-El (Innsbruck, Austria), and Oticon Medical/Neurelec (Vallauris, France).

The temporal information of the speech signal is commonly decomposed into envelope (2–50 Hz), periodicity (50–500 Hz), and temporal fine structure (TFS; 500–10,000 Hz), for instance described by Wouters et al. (2015). The envelope is the slow variations in the speech signal. Periodicity corresponds with the vibrations of the vocal cords, which conveys fundamental frequency (F0) information. TFS is the fast fluctuations in the signal, and contributes to pitch perception, sound localization, and binaural segregation of sound sources.

All stimulation strategies represent high-frequency sounds only by place coding. Moreover, the stimulation rate in every implant is constant, varying between 500 and 3,500 pulses per second for the different manufacturers. Low-frequency sounds can be represented by both temporal and place coding.

In the present study, the consonant and vowel repetition scores and confusions were measured using an NSRT with recorded monosyllabic consonant-vowel-consonant (CVC) and bisyllabic vowel-consonant-vowel (VCV) nonsense words, named nonsense syllables in this article, in an open-set design. By open-set design, we mean that the responses are not made through a forced choice of alternatives, but rather by repetition of what is perceived. The nonsense syllables follow the phonotactic rules of the participants’ native language, which in our case is Norwegian (e.g., Coady and Aslin, 2004). To avoid straining the working memory, each stimulus unit was limited to 1 or 2 syllables (Gathercole et al., 1994). In the following, the rationale for the test design is presented.

Speech perception tests for children with CIs are traditionally performed with live or recorded real words or sentences in quiet or in noise (e.g., Harrison et al., 2005; Zeitler et al., 2012; Ching et al., 2018). Such tests indisputably measure the children’s language skills in addition to their auditory skills.

There are two methods of making speech perception tests more difficult in order for the test subjects not to perform at ceiling. One is to degrade the speech signal by altering its temporal and spectral information, for instance by adding background noise to the test words or applying high- or low-pass filtering. Perception of speech in background noise is more difficult than in quiet due to factors such as diminished temporal coding (Henry and Heinz, 2012). The other method is to use more challenging test units, such as words without lexical meaning, and assess details in the perception of individual speech sounds under optimal listening conditions. The use of an NSRT in quiet allows for directly studying feature information transmission as opposed to tests relying on a degraded speech signal. In real life, listeners are faced with challenging situations similar to NSRTs when they try to catch an unfamiliar name or are confronted with new vocabulary. New and difficult words are perceived as nonsense syllables until they become internalized as meaningful units.

The measurement of consonant and vowel scores in children with CI’s via recorded nonsense syllables has rarely been reported in scientific literature. A systematic review and meta-analysis by Rødvik et al. (2018), found only two studies of this kind (Tyler, 1990; Arisi et al., 2010). Tyler (1990) included five children who were asked to choose between several written alternatives when they identified each nonsense syllable. Their mean (*SD*) age at testing was 8.5 (1.6) years, and they obtained a mean (*SD*) consonant identification score of 30% (13%) (range: 19–50%). The reason for this relatively low score was probably the high age at implantation for these prelingually (*N* = 2) and postlingually (*N* = 3) deaf children [mean (*SD*) = 7.4 (1.9) years]. Arisi et al. (2010) included 45 adolescents with a mean (*SD*) age of 13.4 (2.6) years, who obtained a mean (*SD*) consonant identification score of 53.5 (33.6)%. All participants marked their choices with a pen on printed text.

We chose a test with verbal repetition of the test words, to ensure that the test scores would neither be influenced by the test subjects’ reading or writing ability nor their computer skills, and that they were not required to relate to anything other than their own hearing and speech as well as their own established phoneme inventory. This design provided detailed information about speech perception and listening capacity for acoustic properties.

Furthermore, an open-set test design was chosen, in which the participants did not know which or how many test units would be presented to them. The participants were thus not limited in their responses and would find no external clues when interpreting what they heard. Previous studies have reported robust effects of competition between items in the mental lexicon and of speaker variability in open-set but not in closed-set tests (e.g., Sommers et al., 1997; Clopper et al., 2006). Moreover, open-set test designs have relatively small learning effects compared to closed-set test designs and can therefore be performed reliably at desired intervals (Drullman, 2005, p. 8).

Open-set test designs also have some disadvantages. For example, they often result in lower overall performance than closed-set test designs and may be challenging to use with low-performing adults and young children. Moreover, they require a substantial effort in post-test analysis if each response is to be transcribed phonetically. Alternatively, responses may be scored simply as correct or incorrect for routine-testing in a clinical practice.

**FIGURE 1:**
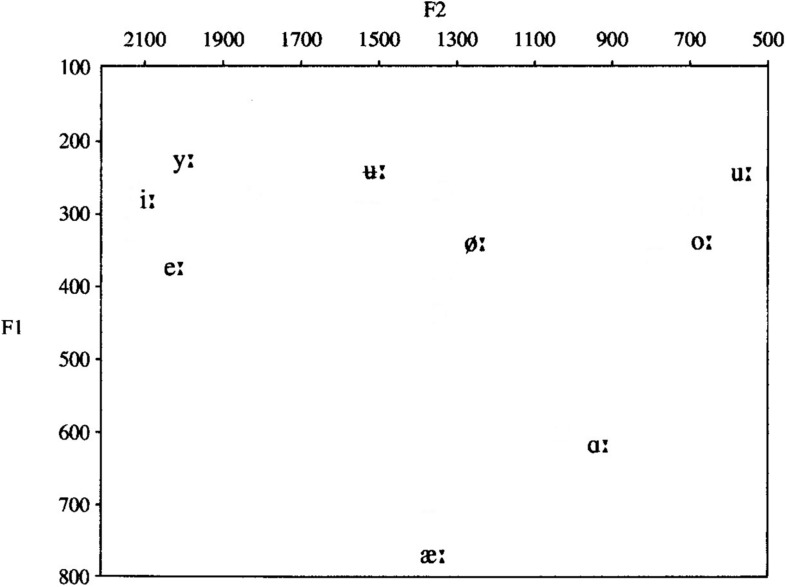
Simplified vowel chart displaying the long Norwegian vowels used in the NSRT, plotted according to the two first formant frequencies, F1 and F2 [modified after Kristoffersen, 2000 (2000, p. 17)].

Norwegian is a Northern Germanic language, belonging to the Scandinavian language group. There is no official common Norwegian pronunciation norm, as oral Norwegian is a collection of dialects, and Norwegians normally speak the dialect of their native region. Norwegian has two lexical tones (except for certain dialects), which span across bisyllabic words and are used as a distinguishing, lexical factor. The tones’ melodies are indigenous to each dialect and are recognized as a dominant and typical prosodic element of the dialect, distinguishing it from other dialects. Norwegian has a semi-transparent orthography, meaning that there is not a consistent one-to-one correspondence between letters and phonemes, like for instance in Finnish, but a much more transparent relation between phonemes and letters than in English (Elley, 1992). In the present study, only speech sounds common for all Norwegian dialects are included; see Table 1 and Figure 1 for an overview.

**TABLE 1:** Simplified IPA chart displaying the speech sounds used in the NSRT.

	**Place of articulation**													
	
	**Bilabial**		**Labiodental**		**Dental**		**Post-alveolar**		**Palatal**		**Velar**		**Glottal**	
							
**Manner of articulation**	**U**	**V**	**U**	**V**	**U**	**V**	**U**	**V**	**U**	**V**	**U**	**V**	**U**	**V**
Stops	[p]	[b]			[t]	[d]					[k]	[g]		
Fricatives			[f]		[s]		[ʃ]			[j]			[h]	
Nasals		[m]				[n]						[ŋ]		
Lateral						[l]								

*U = unvoiced; V = voiced.*

The overall objective of the present study was to measure the perception of speech sounds in well-performing children and adolescents with CIs with an NSRT.

The two sub-objectives were as follows:

Objective 1: To identify the most common vowel and consonant confusions and the most common confusions of the phonetic features voicing, frication, stopping, nasality, and laterality in a sample of well-performing children and adolescents with CIs.

Objective 2: To investigate how age at onset of severe to profound (pre-, peri-, and postlingual) deafness in children and adolescents with CIs influences their confusion of speech sounds and features.

## Materials and Methods

Abbreviations and acronyms are presented in Table 2.

**TABLE 2:** List of acronyms and abbreviations.

**Number**	**Abbreviation/acronym**	**Meaning**
1	ACE	Advanced combination encoder (stimulation strategy from Cochlear)
2	CI	Cochlear implant
3	CIS	Continued interleaved sampling (generic stimulation strategy)
4	CM	Confusion matrix
5	CVC	Consonant-vowel-consonant
6	F0, F1, F2	Fundamental frequency, first formant, and second formant
7	FSP/FS4/FS4-p	Fine structure processing (stimulation strategies from Med-El)
8	HA	Hearing aid
9	HIST	Høgskolen i Sør-Trøndelag (real-word monosyllable test)
10	HL	Hearing level
11	NH	Normal-hearing
12	NSRS	Nonsense syllable repetition score
13	NSRS-C	Nonsense syllable repetition score – consonants
14	NSRS-C_voi_	Nonsense syllable repetition score – voiced consonants
15	NSRS-C_unvoi_	Nonsense syllable repetition score – unvoiced consonants
16	NSRS-C_aCa_	Nonsense syllable repetition score – consonants in the aCa context
17	NSRS-C_iCi_	Nonsense syllable repetition score – consonants in the iCi context
18	NSRS-C_uCu_	Nonsense syllable repetition score – consonants in the uCu context
19	NSRS-C_pre_	Nonsense syllable repetition score – consonants repeated by prelingually deaf
20	NSRS-C_post_	Nonsense syllable repetition score – consonants repeated by postlingually deaf
21	NSRS-V	Nonsense syllable repetition score – vowels
22	NSRS-V_pre_	Nonsense syllable repetition score – vowels repeated by prelingually deaf
23	NSRS-V_post_	Nonsense syllable repetition score – vowels repeated by postlingually deaf
24	NSRT	Nonsense syllable repetition test
25	PTA	Pure-tone average
26	REC	Regional ethical committee
27	T, T_max_, T_rel_	Speech transmission index (absolute, maximum, and relative)
28	TFS	Temporal fine structure
29	VCV	Vowel-consonant-vowel
30	VOT	Voice onset time

### Participants

Informed written consent was obtained from all participants and their legal guardians, according to the guidelines in the Helsinki declaration (World Medical Association [WMA], 2017). The project was approved by the ethical committee of the regional health authority in Norway (REC South East) and by the data protection officer at Oslo University Hospital.

#### Participants With CIs

Thirty-six children and adolescents with CIs (18 girls) participated in this study. Their age range was 5.9–16.0 years [mean (*SD*) = 11.6 (3.0) years]. Oral language was the main communication mode for all participants. The study sample included 29 prelingually and 7 postlingually deaf participants using the CI stimulation strategies FS4 (*N* = 4), FSP (*N* = 7), and CIS + (*N* = 2) from Med-El and ACE (*N* = 23) from Cochlear (abbreviations are explained in Table 2).

The following inclusion criteria were met for all of these participants: minimum 6 months of implant use, more than 3 months since the activation of the second CI (if they had one), and unchanged processor settings for at least the last 2 months. Furthermore, the participants were required to obtain a score of more than 50% on the HIST monosyllable test in free-field (Øygarden, 2009) and to spontaneously pronounce 100% of all the Norwegian speech sounds correctly. Subjects with a contralateral HA were excluded.

All the included participants were enrolled in the CI program at Oslo University Hospital and were recruited for the present study as part of their ordinary follow-up appointments. Individual demographic information is shown in Supplementary Table S1, and individual test results are listed in Supplementary Table S2.

#### Reference Groups

The two reference groups of NH participants were: seventeen 6-year-olds (7 girls; [mean (*SD*) age = 5.9 (0.3) years; range: 5.3–6.3 years]), and twelve 13-year-olds (7 girls; [mean (*SD*) age = 13.0 (0.3) years; range: 12.5–13.3 years]). Six years was an appropriate lower age limit in the reference group, as the majority of children of this age were able to pronounce all the speech sounds correctly in their own dialect. The NH 6-year-olds were mainly recruited from kindergartens near the hospital, and the 13-year-olds were recruited from a primary school nearby.

Normal hearing was confirmed by pure-tone audiometry showing audiometric thresholds at 20 dB (HL) or better on frequencies between 125 and 8,000 Hz. We chose a level of uncertainty of 5 dB, according to the *SDs* of measured audiometric thresholds in a large group of NH listeners in a study by Engdahl et al. (2005). Thus, also children and adolescents with hearing thresholds at 25 dB were included. The middle-ear status of the reference groups was checked with tympanometry and otomicroscopy by an ear, nose, and throat specialist before audiometry.

#### Inclusion Criteria for All Groups

All participants were required to have Norwegian as their native language and to obtain a 100% score on a pronunciation test of all the target speech sounds in the NSRT.

### Test Descriptions

#### The Nonsense Syllable Repetition Test

The NSRT contains the 16 consonant sounds that are common for all Norwegian dialects, [p, t, k, s, ʃ, f, h, b, d, ɡ, ʝ, v, n, m, ŋ, l], and 11 additional consonant sounds that are used in local Norwegian dialects. To avoid dialect background as a confounding factor in our study, only the first-mentioned 16 consonants were included in the analyses, as they were familiar to all participants. The consonants were placed in a bisyllabic VCV context with the three main cardinal vowels in Norwegian, /ɑ:, i:, u:/ (see Supplementary Table S3). Table 1 presents a simplified IPA chart of the included consonants, classified by manner and place of articulation, and by voicing/non-voicing.

The NSRT also contains the nine Norwegian long vowels, [ɑ:, e:, i:, u:, u :, y:, æ:, ø:, ɔ:], presented in a monosyllabic CVC context with /b/ as the chosen consonant (see the vowel chart in Figure 1 and an overview of the nonsense syllables in Supplementary Table S3).

None of the CVC or VCV combinations presented in the test had lexical meaning in Norwegian. Recording and preparation of the test was mainly done with the computer program Praat (Boersma and Weenink, 2018) and is described in Supplementary Data Sheet S1 and Introduction provides the rationale for using a repetition test with nonsense syllables in an open-set design.

#### Real-Word Monosyllable Test

The perception of real-word monosyllables was measured by the HIST monosyllable test in free-field, a test with 50 Norwegian phonetically balanced words, which produces a percent score (Øygarden, 2009). The test words were presented at 65 dB(A), and 1 out of 12 lists was chosen.

#### Pronunciation Test

A sample of “Norsk fonemtest” (Norwegian test of phonemes; Tingleff, 2002) with 28 of its 104 pictures, was used to assess the participants’ ability to pronounce all Norwegian consonants and vowels correctly. The selected test items presented the target phoneme in the medial position to match their position in the NSRT. Only those who obtained a 100% score on this test were included in the study.

### Procedure and Design

The test words were presented from a SEAS 11F-LGWD 4.5” loudspeaker (Moss, Norway), in an anechoic chamber via the computer program SpchUtil, v. 5 (Freed, 2001). The hard disk recorder Zoom H4n (Hauppauge, NY, United States) was used to record the repeated test words and the naming of the pictures. The distance between the loudspeaker and the participants was 1.5 m, and the equivalent sound level in listening position was 65 dB(A).

#### Testing of Children and Adolescents With CIs

The NSRT was conducted by playing the recorded CVC and VCV nonsense syllables in randomized order and recording participants’ verbal repetitions. The participants were exposed to auditory stimuli only and could not rely on lipreading. They were informed that words with no meaning would be presented to them, but they were not given any further details about how many, which words, and in which consonant or vowel context the speech sounds would be presented.

The participants were instructed to repeat what they heard and to guess if they were unsure, in order to achieve a 100% response rate. Each speech stimulus was presented only once, and the participants were not allowed to practice before being tested or provided with feedback during the testing.

The ecological validity of the testing was optimized by having the participants use the everyday settings of their speech processors instead of switching off front-end sound processing, which has been done in similar studies (e.g., Wolfe et al., 2011). The speech processors were quality checked before testing, and new programming was not performed prior to the testing.

Unaided pure-tone audiometry was performed to check for residual hearing, if these results were not present in the patient’s file. Otomicroscopy was performed by an ear, nose, and throat specialist if the participant had residual hearing in one ear or if middle-ear problems were suspected.

Fifty HIST monosyllabic test words in free-field were conducted with all the participants with CIs.

#### Testing of Normal-Hearing Children and Adolescents

The test setup for the NH reference groups corresponded to that for the participants with CIs, except that the HIST monosyllable test was not conducted, because listeners with normal hearing typically perform at the ceiling level on this test.

#### Phonetic Transcription and Scoring

The recordings of the participants’ repetitions were transcribed by two independent, trained phoneticians, who were blind to the purpose of the study and to what kind of participant groups they transcribed. The transcribers performed a broad phonetic transcription of the nonsense syllables in the test, including primary and secondary stress, and lexical tone, but not suprasegmentals.

The transcriptions of the two phoneticians were compared, and in the case of disagreement between the transcribers, the first author listened to the recordings and picked the transcription that he judged to be correct. The mean (*SD*; range) exact percent agreement between the two transcribers was 82.8 (6.6; 66.7–98.2)% for the participants with CIs and 89.2 (7.5; 68.4–100)% for the NH reference groups.

The repetitions of each target speech sound were scored as either correct (1) or incorrect (0). The total scores were calculated by dividing the number of correctly repeated responses by the total number of stimuli, for the consonants, averaged for the three vowel contexts (NSRS-C), for the vowels (NSRS-V), for the consonants in aCa, iCi, and uCu contexts (NSRS-C_iCi_, NSRS-C_aCa_, and NSRS-C_uCu_), and for the voiced and unvoiced consonants averaged for the three vowel contexts (NSRS-C_voi_ and NSRS-C_unvoi_). The consonant and vowel scores for the subgroups of prelingually and postlingually deaf were calculated by dividing the number of correctly repeated responses by the total number of stimuli for each subgroup (NSRS-C_pre_, NSRS-C_post_, NSRS-V_pre_, and NSRS-V_post_). The nonsense syllable repetition score (NSRS) was produced by calculating a weighted mean of NSRS-V and NSRS-C, in which the weights were determined by the number of different vowels (9) and consonants (16) in the test [NSRS = (NSRS-V × 9 + NSRS-C × 16)/25].

### Analysis

The 12 variables mentioned in the previous section (#12–23 in Table 2) were constructed to score the performance on the NSRT for the three groups of participants, and means, medians, and standard deviations were calculated for all variables. The consonant speech features voicing, stopping, frication, nasality, and laterality were examined separately in the analyses. Assumptions of a normal distribution were violated due to checking of the data with the Shapiro–Wilk test, possibly due to a ceiling effect in some of the variables. Therefore, scores from the participants with CIs were compared by the non-parametric Wilcoxon signed rank z test for related samples, for the following variables:

• Voiced and unvoiced consonant scores (NSRS-C_voi_ and NSRS-C_unvoi_).
• The HIST real-word monosyllable score and the NSRS.
• NSRS-C_aCa_, NSRS-C_iCi_, and NSRS-C_uCu_.
• The consonant and vowel scores (NSRS-C and NSRS-V).
• Consonant and vowel scores for the pre- and postlingually deaf (NSRS-C_pre_, NSRS-C_post_, NSRS-V_pre_, and NSRS-V_post_).

Comparisons of NSRS-C and NSRS-V, and NSRS-C_voi_ and NSRS-C_unvoi_, were also performed for the NH 6- and 13-year olds. Correlations were calculated with Spearman’s rho (ρ*).*

Scores on all variables were compared between the CI users and the NH 6-year-olds, and between the NH 6-year-olds and the NH 13-year-olds, with the Mann–Whitney *U* test for independent samples. To determine statistical significance, an alpha (α) level of 0.05 was chosen for all tests.

**FIGURE 2:**
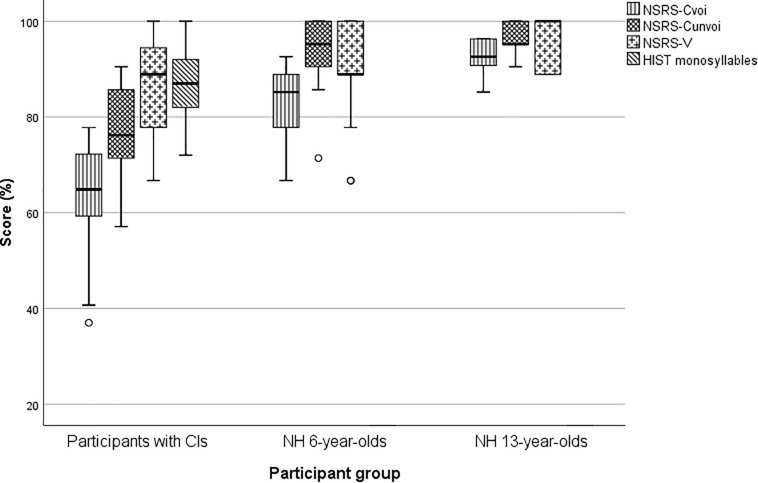
Unvoiced and voiced consonant scores, vowel scores, and monosyllable scores for the three participant groups. The small circles are outliers that represent scores larger than 1.5 times the interquartile range of the box.

Box-and-whiskers were used to display the score distribution for HIST monosyllables, NSRS-V, NSRS-C_unvoi_, and NSRS-C_voi_ for the three participant groups (see Figure 2). All statistical analyses were performed by SPSS v. 24.0 (SPSS Inc., Chicago, IL, United States). A Holm-Bonferroni correction was used to correct for multiple comparisons in all the statistical tests.

#### Information Transmission for Subgroup Comparisons of Speech Sound Features

The speech sound confusions were organized into confusion matrices (CMs). The CM for the consonant confusions was submitted to an information transfer analysis. This method was introduced by Miller and Nicely (1955) and is an application of the information measure by Shannon (1948) to obtain data from a speech repetition task and measure the covariance of input and output in a stimulus-response system. The method produces a measure of mean logarithmic probability. The logarithm is taken to the base 2, and the measure can thus be called the average number of binary decisions needed to specify the input, or the number of bits of information per stimulus. The method has been used in a large number of studies of the speech sound perception of implantees (e.g., Tye-Murray et al., 1990; Tyler and Moore, 1992; Doyle et al., 1995; Sheffield and Zeng, 2012; Yoon et al., 2012).

The advantage of using this unit instead of recognition scores of correct and incorrect repetitions that are measured binarily is that the repetition errors within the same category of speech sounds obtain higher scores than repetition errors between different categories.

The information transmission (T) was calculated with the formula below:

$$T=-\sum_{i}\sum_{j}\frac{n_{i\,j}}{n}\,\log_{2}\frac{\frac{n_{i}}{n}\,\frac{n_{j}}{n}}{\frac{n_{i\,j}}{n}}$$

Here, *i* and *j* are the stimulus number and response number (the column and row numbers of the CM, respectively), *n*_*ij*_ is the cell value, *n*_*i*_ is the row sum, *n*_*j*_ is the column sum, and *n* is the total sum.

The relative transmission, *T*_rel_, is given by *T*_rel_ = *T*/*T*_max_, in which *T*_max_ is the maximum transmission of information. *T*_max_ describes the transmission if all the speech sounds were repeated correctly and no stimulus/response pairs were missing, and *T* is the absolute transmission. *T*_rel_ was calculated for the speech sound feature contrasts voicing versus non-voicing, nasality versus non-nasality, frication versus stopping, and nasality versus the lateral [l] for the subgroups of the prelingually (*N* = 29) and postlingually (*N* = 7) deaf.

**FIGURE 3:**
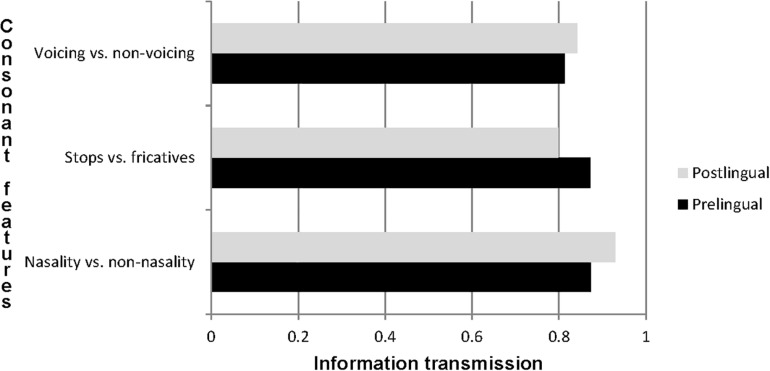
Relative transmission of speech features for pre- and postlingually deaf participants with CIs.

The information transmissions for the subgroups were compared by collapsing the CMs in Table 6 and analyzing them by χ^2^ statistics. Fisher’s exact test was applied if the number in one of the quadrants in the 2 × 2 tables was lower than 5. Our null hypothesis was that the information transmission was equally large for both pre- and postlingually deaf participants. A histogram was constructed to visualize the transmission of speech sound features for the two groups (Figure 3).

## Results

### Study Characteristics

The medians of the three groups of participants are displayed in Table 3, and comparisons of the participants with CIs and

**TABLE 3:** *M, Md*, and *SD* of the study variables for the participants with CIs, the NH 6-year-olds, and the NH 13-year-olds.

	**CI users (*N* = 36)**			**NH 6-year-olds (*N* = 17)**			**NH 13-year-olds (*N* = 12)**		
			
**Variable (%)**	***M* (*SD*)**	***Md***	**Range**	***M* (*SD*)**	***Md***	**Range**	***M* (*SD*)**	***Md***	**Range**
NSRS	75.2 (8.0)	77.3	56.0–89.3	87.6 (5.8)	88.0	72.0–94.7	94.8 (2.0)	94.7	90.7–97.3
NSRS-C	69.6 (8.0)	70.8	50.0–83.3	86.9 (6.1)	87.5	72.9–93.8	94.4 (2.7)	95.8	89.6–97.9
NSRS-C_aCa_	78.0 (8.6)	81.3	56.3–93.8	90.1 (7.3)	87.5	75.0–100	97.9 (3.1)	100	93.8–100
NSRS-C_iCi_	69.3 (12.3)	71.9	25.0–87.5	89.3 (4.8)	87.5	81.3–100	96.4 (5.0)	100	87.5–100
NSRS-C_uCu_	61.5 (13.1)	62.5	31.3–93.8	81.3 (12.1)	87.5	56.3–100	89.1 (3.9)	87.5	81.3–93.8
NSRS-C_voi_	63.9 (10.6)	64.9	37.0–77.8	82.6 (7.5)	85.2	66.7–92.6	92.6 (3.5)	92.6	85.2–96.3
NSRS-C_unvoi_	76.9 (9.3)	76.2	57.1–90.5	92.4 (7.5)	95.2	71.4–100	96.8 (3.1)	95.2	90.5–100
NSRS-C_pre_	69.1 (7.8)	70.8	50.0–81.3	–	–	–	–	–	–
NSRS-C_post_	71.4 (9.0)	70.8	56.3–83.3	–	–	–	–	–	–
NSRS-V	85.2 (10.9)	88.9	66.7–100	88.9 (11.1)	88.9	66.7–100	95.4 (5.7)	100	88.9–100
NSRS-V_pre_	86.2 (10.1)	88.9	66.7–100	–	–	–	–	–	–
NSRS-V_post_	81.0 (13.9)	88.9	66.7–100	–	–	–	–	–	–
HIST monosyllable score	86.9 (6.7)	87.0	72.0–100	–	–	–	–	–	–

the NH 6-year-olds, and of the NH 6-year-olds and the NH 13-year-olds with independent sample Mann–Whitney tests, are displayed in Table 4. The results show, as expected, that the NH 6-year-olds had significantly higher scores than the participants with CIs on all variables, except on the NSRS-V. The comparisons of the medians of the NH 6- and 13-year-olds show a significantly higher score for the 13-year-olds for all variables except NSRS-C_uCu_, NSRS-C_unvoi_, and NSRS-V.

**TABLE 4:** Comparisons of the study variables for the participants with CIs, the NH 6-year-olds, and the NH 13-year-olds.

	**CI users vs.**				**NH 6-year-olds vs.**			
	NH 6-year-olds^*^				**NH 13-year-olds^∗∗^**			
		
**Variable (%)**	***U***	***z***	***p***	***r***	***U***	***z***	***p***	***r***
NSRS	47.0	–4.94	<0.001	0.68	20.5	–0.64	<0.001	0.12
NSRS-C	23.0	–5.41	<0.001	0.74	19.5	–3.73	<0.001	0.69
NSRS-C_aCa_	84.5	–4.30	<0.001	0.59	34.0	–3.17	0.002	0.59
NSRS-C_iCi_	22.5	–5.47	<0.001	0.75	35.0	–3.12	0.002	0.58
NSRS-C_uCu_	85.5	–4.25	<0.001	0.58	60.0	–1.96	0.050^∗∗∗^	0.36
NSRS-C_voi_	40.0	–5.10	<0.001	0.70	18.5	–3.76	<0.001	0.70
NSRS-C_unvoi_	61.0	–4.70	<0.001	0.65	65.5	–1.69	0.091	0.31
NSRS-V	264.5	–0.83	0.404	0.11	68.5	–1.62	0.105	0.30

^*^The columns show the results of comparisons of means with the Mann–Whitney independent samples *U*-test between participants with CIs and NH 6-year olds. ^∗∗^The columns show the results of comparisons of means with the Mann–Whitney independent samples *U*-tests between NH 6- and 13-year-olds. ^∗∗∗^The comparison was non-significant after adjusting for multiple testing. The medians and sample sizes that were used in the analyses can be found in Table 3.

In Table 5 the medians for the three groups of participants were compared with Wilcoxon’s signed rank test and Mann-Whitney’s U-test, and furthermore, correlations between the HIST score and NSRS-C_voi_, NSRS-C_unvoi_, and NSRS-V were shown. For the children with CIs, statistically significant differences were found for NSRS-V versus NSRS-C, NSRS-C_unvoi_ versus NSRS-C_voi_, NSRS-C_aCa_ versus NSRS-C_iCi_, and NSRS-C_aCa_ versus NoSRS-C_uCu_. No statistically significant differences were found for NSRS-C_iCi_ versus NSRS-C_uCu_, NSRS-C_pre_ versus NSRS-C_post_, and NSRS-V_pre_ versus NSRS-V_post_. For the NH participants, no statistically significant difference was found, except for the comparison of NSRS-C_unvoi_ and NSRS-C_voi_ for the NH 6-year-olds.

**TABLE 5:** Comparisons of the study variables for the participants with CIs, the NH 6-year-olds, and the NH 13-year-olds.

**Comparison**	**Participant group**	**Statistical test**	**ρ**	***U***	***z***	***p***	***r***
HIST vs. NSRS-C_unvoi_	CI	S	0.26	–	–	0.13	–
HIST vs. NSRS-C_voi_	CI	S	0.41	–	–	0.013^*^	–
HIST vs. NSRS-V	CI	S	0.18	–	–	0.31	–
HIST vs. NSRS	CI	W	–	–	−4.90	<0.001	0.82
NSRS-V vs. NSRS-C	CI	W	–	–	−5.12	<0.001	0.85
	NH6	W	–	–	−0.78	0.43	0.19
	NH13	W	–	–	−0.32	0.75	0.09
NSRS-C_unvoi_ vs. NSRS-C_voi_	CI	W	–	–	−4.46	<0.001	0.74
	NH6	W	–	–	−3.15	0.002	0.76
	NH13	W	–	–	−2.60	0.009^*^	0.75
NSRS-C_aCa_ vs. NSRS-C_iCi_	CI	W	–	–	−3.96	<0.001	0.66
	NH6	W	–	–	−0.18	0.86	0.04
	NH13	W	–	–	−0.97	0.33	0.27
NSRS-C_aCa_ vs. NSRS-C_uCu_	CI	W	–	–	−4.75	<0.001	0.79
	NH6	W	–	–	−2.64	0.008^*^	0.64
	NH13	W	–	–	−2.99	0.003^*^	0.86
NSRS-C_iCi_ vs. NSRS-C_uCu_	CI	W	–	–	−2.76	0.006^*^	0.46
	NH6	W	–	–	−2.51	0.012^*^	0.61
	NH13	W	–	–	−2.72	0.006^*^	0.79
NSRS-C_pre_ vs. NSRS-C_post_	CI	M-W U	–	85.00	−0.66	0.51	0.11
NSRS-V_pre_ vs. NSRS-V_post_	CI	M–W *U*	–	80.00	−0.91	0.36	0.15

CI = cochlear implant; NH6 = NH 6-year-olds; NH13 = NH 13-year-olds; S = Spearman’s correlation test; W = Wilcoxon’s signed rank test; M–W *U* = Mann–Whitney’s *U*-test for independent samples. ^*^Not significant after adjusting for multiple testing. The medians and sample sizes that were used in the analyses can be found in Table 3.

### Consonant Confusions

Tables 6, 7 show the CMs for the 16 consonants in aCa, iCi, and uCu contexts for the 36 participants with CIs. The consonants are grouped primarily as voiced and unvoiced and secondarily according to manner of articulation. Of the consonant stimuli, 223 (12.9%) were repeated as consonant clusters or as consonants other than the ones listed in the CM and were excluded from the analyses. These are listed in the unclassified category of the CM.

**TABLE 6:** Confusion matrix for 36 participants with CIs; consonants in the aCa, iCi, and uCu contexts added together.

			**Response**																	
				
			**Unvoiced**							**Voiced**										
						
**Stimulus**			**S**			**F**				**S**			**F**		**Na**			**L**		
									
			**/p/**	**/t/**	**/k/**	**/s/**	**/ʃ/**	**/f/**	**/h/**	**/b/**	**/d/**	**/g/**	**/j/**	**/v/**	**/n/**	**/m/**	**/ŋ/**	**/l/**	**U**	**N**
Unvoiced	S	/p/	86	6				10		3	1								2	108
		/t/		84	4						2								18	108
		/k/	5	4	89							1							9	108
	F	/s/				93	5	4											6	108
		/ʃ/				13	75												20	108
		/f/	1			14	13	73	4										3	108
		/h/					3	13	81		2		1						8	108
Voiced	S	/b/	13	1	1				1	66	11			4					11	108
		/d/		6							85	3							14	108
		/g/			9				1		2	90		2					4	108
	F	/j/							2			2	88						16	108
		/v/							1		1	1		83		1			21	108
	N	/n/													77	9		2	20	108
		/m/													29	66	2	1	10	108
		/ŋ/											1		43	21	16	4	23	108
	L	/l/										1		1	2			66	38	108
																			Total sum	1, 728

*S = stops; F = fricatives; Na = nasals; L = lateral [l]; U = unclassified speech sounds and consonant clusters.*

**TABLE 7:** Confusion matrix of consonant repetitions for participants with CIs, collapsed with regard to manner and place of articulation (percentage of correctly repeated stimulus features in each cell).

			**Response (%)**																		
			
			**Unvoiced**							**Voiced**											
							
			**S**			**F**				**S**			**F**		**Na**			**L**			
											
**Stimulus**			**/p/**	**/t/**	**/k/**	**/s/**	**/ʃ/**	**/f/**	**/h/**	**/b/**	**/d/**	**/g/**	**/j/**	**/v/**	**/n/**	**/m/**	**/ŋ/**	**/l/**	**U**	**Sum (%)**	**N**
Unvoiced	S	/p/																			
		/t/	85.8			3.1				2.2									9.0	100	324
		/k/																			
	F	/s/	0.2			90.5				0.5			0.2						8.6	100	432
		/ʃ/																			
		/f/																			
		/h/																			
Voiced	S	/b/																			
		/d/	9.3			0.6				79.3			1.9						9.0	100	324
		/g/																			
	F	/j/				1.4				1.9			79.2		0.5				17.1	100	216
		/v/																			
		/n/																			
	N	/m/											0.3		81.2			2.2	16.4	100	324
		/ŋ/																			
	L	/l/								0.9			0.9		1.9			61.1	35.2	100	108
																				Total sum	1, 728

*S = stops; F = fricatives; Na = nasals; L = lateral [l]; U = unclassified speech sounds and consonant clusters.*

The consonant CM in Table 6 shows a devoicing bias for the stops. Unvoiced consonants are in general most frequently confused with other unvoiced consonants and voiced consonants are most frequently confused with other voiced consonants, except for the voiced stops, which are frequently repeated as unvoiced stops. Furthermore, there are highly populated clusters of correct repetitions around voiced and unvoiced stops, voiced and unvoiced fricatives, and nasals.

Table 7 shows that the highest proportion of correct repetitions was within the manner-groups of unvoiced fricatives; 90.5% of these were repeated as the same, or as another unvoiced fricative, and of unvoiced stops; 85.8% were repeated as the same, or as another unvoiced stop. Among the nasals, 81.2% were repeated as the same, or as another nasal, among the voiced fricatives, 79.2% were repeated as the same, or as another voiced fricative, and among the voiced stops, 79.3% were repeated as the same, or as another voiced stop. The highest proportion of consonant confusions was found for the lateral [l], with a correct score of only 61.1%.

**FIGURE 4:**
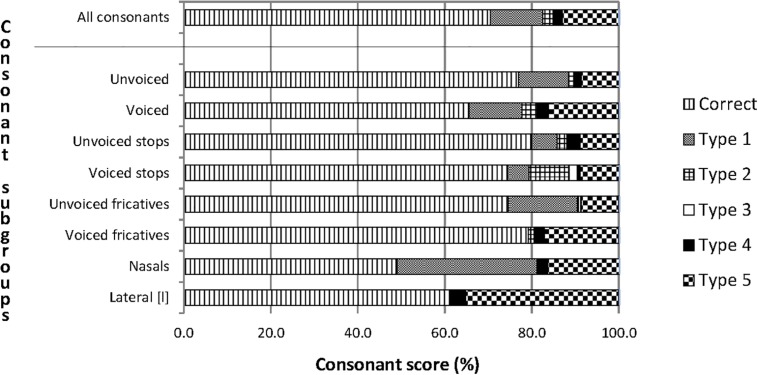
Percentages of correct consonant repetitions and of five types of consonant confusions for participants with CIs. The upper bar describes the complete material of consonant confusions and the eight bars below the horizontal line describe subsets of the material. The units on the horizontal axis are the percentage scores of correct and incorrect repetitions. The bars with a horizontal pattern visualize correct repetitions. Type 1 is confusion between consonants with the same manner and the same voicing. Type 2 is confusion between consonants with the same manner and the opposite voicing. Type 3 is confusion between consonants with a different manner and opposite voicing. Type 4 is confusion between consonants with a different manner and the same voicing. Type 5 is unclassified confusions.

The correct repetition scores of the categories of speech features in Figure 4 ranged from 60% to 80%, except for the nasals, which had a score slightly below 50%. The most common confusions were between consonants with the same manner and same voicing (Type 1 confusions). The least common confusions were between consonants with a different manner and opposite voicing (Type 3 confusions). The number of unclassified confusions, which includes consonant clusters and consonant sounds other than the stimuli, was also substantial, particularly for the lateral [l].

The NH participants repeated almost all the consonants correctly, as shown in Supplementary Tables S4, S5, S7, and S8. However, we observed an important exception for the 6-year-olds: 10 (19.6%) of the /ŋ/ stimuli were confused with /m/. The 13-year-olds also had an unexpectedly high number of misperceptions of /ŋ/ (7; 19.4%).

### Vowel Confusions

Only two cases of unclassified vowels were found among the nine vowels in the bVb context for the 36 participants with CIs (Table 8). An [i:]-[y:] perception bias was revealed; [y:] was more frequently repeated as [i:] (67%) than as [y:] (31%).

**TABLE 8:** Confusion matrix of vowel repetitions in the bVb context for participants with CIs.

	**Response**										
		
**Stimulus**	**/bɑ:b/**	**/be:b/**	**/bi:b/**	**/bu:b/**	**/bu:b/**	**/by:b/**	**/bæ:b/**	**/bø:b/**	**/bɔ:b/**	**U**	**N**
/bɑ:b/	35									1	36
/be:b/		35	1								36
/bi:b/			36								36
/bu:b/				36							36
/bu:b/			2		30	4					36
/by:b/			24		1	11					36
/bæ:b/	1						35				36
/bø:b/		2	1		5	1		26		1	36
/bɔ:b/				1					35		36
									Total sum		324

*U = unclassified.*

The CMs for the NH children and adolescents (Supplementary Tables S6, S9) show that almost all vowels were repeated correctly. The vowel CM for the 6-year-olds in Supplementary Table S6 shows some randomly distributed errors, in addition to 6 (35%) of the /y:/ stimuli repeated as either /i:/ or /u:/. There were fewer vowel misperceptions for the 13-year-olds than for the 6-year-olds, but even so, 3 (25%) of the /y:/ stimuli were repeated as /i:/, as displayed in Supplementary Table S9.

### Perception of Consonant Features Compared by Information Transmission and Chi Square Statistics Between the Pre- and Postlingually Deaf

Figure 3 shows that nasality versus non-nasality had the highest information transmission, and voicing versus non-voicing had the lowest. The information transmission of speech features did not display large differences between pre- and postlingually deaf participants.

Chi square testing showed no statistically significant differences between the transmission of voicing and non-voicing (χ^2^ = 1.16; *p* = 0.28), nor between the transmission of nasality and non-nasality (χ^2^ = 0.41; *p* = 0.52), nor between the transmission of stops and fricatives (χ^2^ = 1.12; *p* = 0.29). Supplementary Table S10 displays the three 2 × 2 matrices that these analyses are based on.

## Discussion

The objective of this study was to assess the effectiveness of CIs by obtaining a measure of speech sound confusions in well-performing children and adolescents with CIs, using an NSRT, and to investigate whether the perception of speech features differs between the pre- and postlingually deaf. The study was cross-sectional, and it included 36 participants with CIs and 2 reference groups (17 NH 6-year-olds and 12 NH 13-year-olds).

An important finding was that unvoiced consonants were significantly less confused than voiced consonants for the participants with CIs. Moreover, there was a devoicing bias for the stops; unvoiced stops were confused with other unvoiced stops and not with voiced stops, and voiced stops were confused with both unvoiced stops and other voiced stops. Another major finding was that there was no significant difference between the perception of speech sound features for pre- and postlingually deaf CI users.

A central issue when assessing consonant confusions in participants with CIs is to investigate the underlying reasons. Are the confusions caused by limitations in the implants, are they due to immature cognitive development, or can they be explained by other factors? The difference between the NSRS and the HIST real-word monosyllable score suggests that the participants with CIs rely substantially on their language proficiency and the top-down processing introduced by lexical content present in real-word stimulus material. The finding is in line with a study on NH individuals by Findlen and Roup (2011), who investigated dichotic speech recognition performance for nonsense and real-word CVC syllables, and found that performance with nonsense CVC syllables was significantly poorer. Findlen and Roup’s study is to the authors’ knowledge the only previous investigation of recognition differences between real-word and nonsense CVC syllable stimuli that have similar phonetic content but differ in lexical content.

The moderate correlation between NSRS-C_voi_ and HIST monosyllables suggests that problems with perceiving the real-word monosyllables could partly be explained by difficulties in perceiving the voiced consonants.

### The Results of the Participants With CIs Related to Those of the NH Reference Groups

As expected, the scores on the NSRT were higher for the NH 13-year-olds than for the NH 6-year-olds for all variables. However, the differences were not significant for NSRS-C_uCu_, NSRS-C_unvoi_, and NSRS-V, probably because NH 13-year-olds usually have a more developed phonemic lexicon and higher phonemic awareness, or because of age-related differences in attentiveness during the task. We compared the scores of the participants with CIs only to those of the NH 6-year-olds, as these two groups are closest in hearing age. Significant differences were found between the groups of NH 6-year-olds and CI users for all variables except for the NSRS-V, which was just as high for both groups. This may be due to the long duration and high energy of the vowels in the NSRT.

For the NH groups, there were no statistically significant differences in any of the comparisons, except for unvoiced versus voiced consonant score for the NH 6-year-olds. Since this difference was not found for the NH 13-year-olds, this can probably be explained by language immaturity and fatigue.

For the participants with CIs, the difference between voiced and unvoiced consonant scores seems to be mostly due to the fact that unvoiced stops in Norwegian, /p, t, k/, are strongly aspirated and hence have a substantially longer voice onset time (VOT)^2^ than the voiced stops, /b, d, ɡ/ (Halvorsen, 1998). For both CI users and the NH 6-year-olds, the low, voiced consonant score is likely due to the nasals, /m, n, ŋ/, being confused with one another, and by /l/ having a low recognition score.

### The Most Common Confusions of Consonants and Vowels for Participants With CIs

Most consonant confusions observed in the present study can be explained by acoustic similarity in manner and voicing, a conclusion that has also been reached in many previous studies (e.g., Fant, 1973; Dorman et al., 1997; Dinino et al., 2016).

A bias toward unvoiced stops was found, a phenomenon that only occurred for the CI group and hence probably is implant related. This may be related to two main issues: (1) implants convey the F0 in voiced sounds rather poorly due to missing temporal information in the electrical signal for most implant models and to the electrode’s insertion depth possibly being too shallow to cover the whole cochlea (Hamzavi and Arnoldner, 2006; Svirsky et al., 2015; Caldwell et al., 2017) and (2) the VOT makes the unvoiced stops much easier to perceive than the voiced stops due to the aspirated pause between the stop and the following vowel in the VCV syllables.

The subgroups of voiced and unvoiced stops can be distinguished by the presence of a silent gap in the unvoiced stops (Lisker, 1981). For Norwegian unvoiced stops, as for unvoiced stops in most Germanic languages, aspiration is a salient feature: a distinct final auditory breathy pause that is created by closing the vocal cords from a maximally spread position, lasting longer than the occluded phase of the stop articulation (Kristoffersen, 2000). Stops can be difficult to identify, since they are very short and unvoiced stops have little acoustic energy. In identifying stops, CI users usually rely considerably on the spectral properties of the surrounding vowels, such as locus and length of the formant transitions, spectral height and steepness, and VOT (Välimaa et al., 2002).

Moreno-Torres and Madrid-Cánovas (2018) found a voicing bias for the stops for children with CIs, which is the opposite of the results of the present study. Their study design is, however, considerably different from the present study, as the children were Spanish-speaking and were tested with added, speech-modulated noise, which may create a perception of voicing. Also, Spanish does not have aspiration as a salient feature of unvoiced stops, as Norwegian has. Studies with English and Flemish participants have found a devoicing bias similar to our study (e.g., van Wieringen and Wouters, 1999; Munson et al., 2003).

The least correctly repeated consonant was the lateral [l], which elicited many confusions in the unclassified category of the CMs and had the largest difference in correct scores between the participants with CIs and the NH 6-year-olds. Since all the NH participants were recruited from the same dialect area, Standard East Norwegian, many of them confused [l] with [ɭ], which is also part of their speech sound inventory. Remarkably, [l] was almost never confused with the nasals for any of the participant groups.

The nasals, [m, n, ŋ], were often confused with one another by the participants with CIs, and this – together with the [l]-confusions – comprise most of the difference between the NSRS-C_voi_ and NSRS-C_unvoi_. It seems that nasality adds a new obstacle to consonant recognition. This may be due to the prominence of low frequencies around 250 Hz in the nasals’ spectrum; the nasal murmur, also called the nasal formant (F1). The CIs render low frequencies rather poorly compared to high frequencies (Caldwell et al., 2017; D’Alessandro et al., 2018). Perceptual experiments with NH listeners have shown that nasal murmur and the formant transitions are both important for providing information on place of articulation (e.g., Kurowski and Blumstein, 1984). The transitions of F2 are particularly important; [m] is preceded or succeeded by an F2 transition toward a lower frequency, [n] provides little transition change, and [ŋ] is preceded or succeeded by an F2 transition toward a higher frequency.

Although the NH 6- and 13-year-olds perceived almost all consonants and vowels correctly, they confused /ŋ/ with /m/ in 19.6 and 19.4% of the cases, respectively. This confusion was almost exclusively found in the uCu-context. The reason for this tendency might be twofold. First, the tongue body is very retracted for the Norwegian [u:], with a narrow opening of the mouth and in a position close to the tongue position of [ŋ], making the formant transition audibly indistinct. Second, the listeners might primarily be focused on recognizing letters when performing this type of task. There is no unique letter in Norwegian rendering the speech sound [ŋ], and participants may not on the spur of the moment consider this speech sound an alternative, and instead decide on the one that they find acoustically more similar to the other nasals, [m] and [n], which both correspond to single letters of the alphabet.

The most prevalent vowel confusion for the participants with CIs was [y:] perceived as [i:]. The main reason for this confusion is probably that the F1s of these vowels are low (∼250 Hz) and almost coinciding, and the F2 of [i:] is only slightly higher than of [y:]. These vowels are thus closely located in the vowel chart in Figure 1. However, [i:] was never perceived as [y:], probably because [i:] in Norwegian is about 10 times more prevalent than [y:] (Øygarden, 2009, p. 108), and when in doubt, the participants would be likely to choose the most common of the two speech sounds.

Vowels are known to be more easily perceived than consonants, due to their combination of high intensity and long duration. Norwegian vowels are distinguishable by F1 and F2 alone, as opposed to vowels in other languages, which may also be distinguished by higher formants. Vowels are never distinguished by F0.

### Comparison Between the Pre- and Postlingually Deaf Participants

Between the pre- and postlingually deaf participants, we found no significant differences for the consonant and vowel scores, and no significant differences for the speech feature contrasts voicing versus non-voicing, nasality versus non-nasality, and stopping versus frication. All but three participants were provided with CIs in their optimal (*N* = 28) or late (*N* = 5) sensitive period. Four of the prelingually deaf participants who received CIs in their late sensitive period had used bilateral HAs and developed language in the period between onset of deafness and implantation, and their auditory pathways had presumably been effectively stimulated in this period.

For postlingually deaf CI users, the vowel formants conveyed by the implant tend to be misplaced in the cochlea compared to its natural tonotopy. This may be a reason why acoustically similar vowels are more easily confused for the CI users than for the NH listeners.

The mechanisms of brain plasticity and the consequences of age at onset of deafness (pre-, peri-, and postlingual) are important factors for both auditory and linguistic development. Buckley and Tobey (2011) found that the influence of cross-modal plasticity on speech perception ability is greatly influenced by age at acquisition of severe to profound (pre- or postlingual) deafness rather than by the duration of auditory deprivation before cochlear implantation. In our study, brain plasticity at implantation may be a more relevant prognostic factor for the development of speech perception skills than age at onset of deafness, because of the large individual variations in age at implantation and HA use before implantation.

### The Impact of Vowel and Consonant Context on Recognition

The results of the perception of consonants in different vowel contexts indicated that formant transitions played a larger role for the participants with CIs than for the NH participants, since the influence of vowel context on the consonant score was statistically significant for the CI group but not for the NH groups. This is in accordance with Donaldson and Kreft (2006), who found that the average consonant recognition scores of adult CI users were slightly but significantly higher (6.5%) for consonants presented in an aCa or uCu context than for consonants presented in an iCi context. The vocal tract is more open for [ɑ:] than for [i:] and [u:], making the formant transition more pronounced and the consonants therefore more easily perceptible. The Norwegian [u:] is much more retracted than the English [u:], and thus closer to the velar speech sounds, making their formant transitions more challenging to perceive.

The nine long vowels were presented in only one consonant context, with /b/, as vowel perception is based on steady-state formants rather than on formant transitions.

### Inclusion Criteria and Test Design

By only including well-performing participants with CIs (score above 50% on the HIST monosyllable test and 100% correct spontaneous pronunciation score of all the Norwegian speech sounds), we were able to reveal systematic details in speech sound confusions. If poorer-performing participants with CIs had been included, a great deal of noise would have been added to the CMs, as the unclassified category would have become much larger.

In the present study, other higher language skills are of minor importance, as the NSRT is limited to speech sounds and syllables. We therefore had no inclusion criterion regarding language skills. Since the participants with CIs and the NH 6-year-olds had a similar mean hearing age, some perception problems may be related to their developmental stage in speech perception ability, in addition to being implant related.

As our study required that the participants respond verbally, a closed-set test was not a practical option. Moreover, we consider an open-set test design to be more ecologically valid than a closed-set test design, as repetition of unknown syllables is a common activity for children and one with which they are familiar when acquiring new vocabulary in their everyday life.

### Limitations and Strengths

As expected, we obtained ceiling effects on both the vowel and consonant scores for the NH reference groups. For the participants with CIs, there were ceiling effects only on the vowel scores. This explains lack of statistical significance in many of the comparisons, and is in line with previous studies. For instance, Rødvik et al. (2018) have shown that NSRTs rarely result in ceiling effects when measuring consonant perception for CI users but may do so for vowel perception. It is well known that vowels are easier to perceive than consonants, due to longer duration and higher intensity. All nine Norwegian vowels exist in a long and a short version, and in the NSRT, only long vowels were included, making them audibly very distinct.

An important reason for the ceiling effect on the vowel and HIST scores for the participants with CIs is probably our criterion of only including well-performing CI users who had scores above 50% on HIST. The ceiling effect on the HIST score has probably also weakened the correlations with consonant and vowels scores in the CI users.

Since the test lists of the NSRT counted as many as 90 CVC and VCV words, fatigue and lack of concentration may have influenced the results, especially for the younger children. We randomized the word order to prevent the same words from always appearing at the end of the test list and thus avoiding systematic errors.

This study used a convenience sample due to a limited time window for recruiting participants, who were assessed in conjunction with their regular CI checkup. This design has limitations as far as internal matching regarding, for instance age, gender, age at onset of deafness, duration of implant use, age at implantation, or implant model is concerned. Using a convenience sample may, however, also be considered a strength, as the participants represent a completely random sample of Norwegian-speaking children with CIs, since all implanted children in Norway have received their CI at the same clinic, Oslo University Hospital.

The two groups of pre- and postlingually deaf participants are very different in size, and the participants are very different with regard to level of hearing loss after onset of deafness, HA use before implantation, and age at implantation. Ideally, these factors should have been controlled for, so the evidence present to compare these groups may therefore have been weak.

### Recommendations for Future Research and Clinical Use

This study provides information regarding typical misperceptions of speech sounds in participants with CIs, which may be useful as a basis for further research, focusing on its consequences for CI programming. The information will also be very useful when planning listening and speech therapy for the implantees.

The study might also be used as a basis for the development, validation, and norming of a simplified version of the NSRT to be included in the standard test battery in audiology clinics. Children with CIs tested regularly with the NSRT would be provided with individual feedback on what needs to be targeted in the programming of their CIs and in their listening therapy sessions. Pre- and post-testing with the NSRT can be used as a quality control tool of the programming. A clinical NSRT would also meet the increasing challenge of assessing speech perception in patients with different language backgrounds, as it can be adjusted for different languages by modifying it to only include speech sounds existing in a particular language.

A close examination of the CMs of each individual CI user may possibly be employed when deciding whether to reprogram the CIs or simply adjust the approach in listening therapy, since speech sounds within the same manner-group in the CMs are in general more acoustically similar than speech sounds in different manner groups. Hence, a rule-of-thumb may be that in case of confusions within the same manner-group, start with listening therapy, and in case of confusions between two manner-groups, reprogramming of the implant may be useful.

## Conclusion

For the participants with CIs, consonants were mostly confused with consonants with the same voicing and manner. In general, voiced consonants were more difficult to perceive than unvoiced consonants, and there was a devoicing bias for the stops. The vowel repetition score was higher than the consonant repetition score. Additionally there was a [i:]-[y:] confusion bias, as [y:] was perceived as [i:] twice as often as [y:] was repeated correctly.

The subgroup analyses showed no statistically significant differences between consonant repetition scores for the pre- and postlingually deaf participants.

Although the children with CIs obtained scores close to 100% on vowels and real-word monosyllables, none of them obtained scores for voiced consonants above 78%. This is likely to be related to limitations in CI technology for the transmission of low-frequency sounds, such as insertion depth of the electrode and ability to convey temporal information.

## Author’s Note

Preliminary results from this study were presented at the CI conference: CI 2017 Pediatric 15th Symposium on Cochlear Implants in Children, in San Francisco, United States, July 26–29, 2017.

## Data Availability

The datasets generated for this study are available on request to the corresponding author.

## Ethics Statement

The study was approved by the Regional Ethical Committees for Medical and Health Research Ethics – REC South East, Oslo, Norway. This study was carried out in accordance with the recommendations of “helseforskningsloven” (Health Research Law) §9, §10, §11, and §33, and cf. “forskningsetikkloven” (Research Ethics Law) §4, approved by the REC South East, with written informed consent from all subjects. All subjects gave written informed consent in accordance with the Declaration of Helsinki. The protocol was approved by the REC South East. Additional considerations regarding vulnerable populations such as minors: the speech perception testing of the children included in the project implied no risk for them, and no additional measures were necessary.

## Author Contributions

AR designed the study, analyzed the data, and wrote the manuscript. OT was responsible for the analyses and for technical matters regarding the CI, JT was responsible for methodological, structural, and linguistic matters, OW was responsible for audiological and educational matters, IS was responsible for phonetic and speech therapeutic matters, and JS was responsible for study design and medical matters. All authors discussed the results and suggested revisions of the manuscript at all stages.

## Conflict of Interest Statement

The authors declare that the research was conducted in the absence of any commercial or financial relationships that could be construed as a potential conflict of interest.

## Footnotes

1 PTA is defined as average hearing loss on the frequencies 1,000, 2,000, 3,000, and 4,000 Hz, according to the National Institute for Occupational Safety, and Health [NIOSH] (1996).

2 VOT is the time between air release and vocal-cord vibration.
