# Supplementary material for: Consonant and Vowel Confusions in Well-Performing Children and Adolescents With Cochlear Implants, Measured by a Nonsense Syllable Repetition Test
Source: Front Psychol. 2019 Aug 14;10:1813. doi: 10.3389/fpsyg.2019.01813 (PMC6702790; doi:10.3389/fpsyg.2019.01813)
Supplement: Supplementary file 1 [file Table_1.docx]

| **Table S1 \| Demographics of participants with CIs** | | | | | | | | | | | | |
| --- | --- | --- | --- | --- | --- | --- | --- | --- | --- | --- | --- | --- |
| **Participant no.** | | **Age (Years)** | | | **Gender (Male/ Female)** | **Age at  implantation  (Years)** | **Duration of implant use (Years)** | **°Implant model** | **Stimulation strategy** | **Modality  (1CI—1/  2CI—2)** | **Pre- or postlingually deaf at implantation** | |
| 1 | | 7.5 | | | Female | 4.0 | 3.4 | Nucleus CI512 | ACE | 2 | Post- | |
| 2 | | 11.5 | | | Female | 3.2 | 8.2 | Nucleus CI24RE(CA) | ACE | 2 | Pre- | |
| 3 | | 11.6 | | | Male | 1.0 | 10.5 | Nucleus CI24RE(CS) | ACE | 2 | Pre- | |
| 4 | | 11.1 | | | Male | 1.6 | 9.4 | Pulsar ci100 | FSP | 2 | Pre- | |
| 5 | | 16.0 | | | Female | 2.6 | 13.2 | Nucleus CI24R(ST) | ACE | 2 | Pre- | |
| 6 | | 13.0 | | | Male | 1.6 | 11.2 | Nucleus CI24R(CS) | ACE | 2 | Pre- | |
| 7 | | 9.1 | | | Male | 0.8 | 8.2 | Nucleus CI24RE(CA) | ACE | 2 | Pre- | |
| 8 | | 10.3 | | | Male | 5.1 | 5.1 | Nucleus CI24RE(CA) | ACE | 2 | Pre- | |
| 9 | | 10.2 | | | Female | 4.2 | 5.1 | Sonata i100 | FSP | 2 | Pre- | |
| 10 | | 10.6 | | | Female | 1.4 | 9.1 | Pulsar ci100 | FSP | 2 | Pre- | |
| 11 | | 11.7 | | | Female | 1.0 | 10.6 | Combi 40+ | FSP | 2 | Pre- | |
| 12 | | 15.8 | | | Male | 2.4 | 13.3 | Nucleus CI24M | ACE | 2 | Pre- | |
| 13 | | 14.0 | | | Female | 5.1 | 8.8 | Nucleus CI24RE(CA) | ACE | 2 | Pre- | |
| 14 | | 9.4 | | | Female | 0.5 | 8.8 | Nucleus CI24RE(CA) | ACE | 2 | Pre- | |
| 15 | | 15.3 | | | Female | 3.7 | 11.5 | Combi 40+ | CIS+ | 2 | Post- | |
| 16 | | 8.2 | | | Female | 2.6 | 5.5 | Nucleus CI24RE(CA) | ACE | 2 | Post- | |
| 17 | | 5.9 | | | Female | 1.7 | 4.1 | Nucleus CI512 | ACE | 2 | Pre- | |
| 18 | 15.0 | | | Male | | 7.8 | 7.1 | Nucleus CI24RE(CA) | ACE | 2 | Post- | |
| 19 | 12.4 | | Female | | | 11.7 | 0.6 | Nucleus CI24RE(CA) | ACE | 1 | Post- | |
| **°** Combi 40+, Pulsar ci100, Sonata i100, and Concerto Mi1000 are manufactured by Med-El. Nucleus CIxx is manufactured by Cochlear. The participants are ordered by their test date. | | | | | | | | | | | | |
| (Continued on the following page) | | | | | | | | | | | | |
| 20 | 8.8 | | Male | | | 0.6 | 8.1 | Pulsar ci100 | FS4 | 2 | Pre- | |
| 21 | 13.1 | | Male | | | 1.3 | 11.7 | Combi 40+ | CIS+ | 2 | Pre- | |
| 22 | 6.4 | | Male | | | 1.2 | 5.0 | Nucleus CI512 | ACE | 2 | Pre- | |
| 23 | 14.9 | | Male | | | 6.7 | 8.1 | Pulsar ci100 | FSP | 2 | Pre- | |
| 24 | 8.1 | | Female | | | 1.0 | 7.0 | Sonata ti100 | FS4 | 2 | Pre- | |
| 25 | 13.0 | | Female | | | 3.6 | 9.3 | Pulsar ci100 | FSP | 2 | Pre- | |
| 26 | 10.8 | | Male | | | 1.7 | 9.0 | Nucleus CI24RE(CA) | ACE | 2 | Pre- | |
| 27 | 14.7 | | Female | | | 2.4 | 12.2 | Nucleus CI24R(CS) | ACE | 2 | Pre- | |
| 28 | 9.5 | | Female | | | 3.0 | 6.4 | Nucleus CI24RE(CA) | ACE | 2 | Pre- | |
| 29 | 10.1 | | Male | | | 2.6 | 7.4 | Nucleus CI24RE(CA) | ACE | 2 | Pre- | |
| 30 | 15.7 | | Female | | | 11.7 | 3.9 | Nucleus CI512 | ACE | 2 | Post- | |
| 31 | 6.1 | | Male | | | 0.9 | 5.1 | Nucleus CI512 | ACE | 2 | Pre- | |
| 32 | 8.7 | | Female | | | 0.7 | 7.9 | Nucleus CI24RE(CA) | ACE | 2 | Pre- | |
| 33 | 14.6 | | Male | | | 4.8 | 9.8 | Pulsar ci100 | FS4 | 2 | Post- | |
| 34 | 15.2 | | Male | | | 2.1 | 13.0 | Combi 40+ | FSP | 2 | Pre- |  |
| 35 | 13.9 | | Male | | | 3.2 | 10.5 | Pulsar ci100 | FS4 | 2 | Pre- |  |
| 36 | 14.8 | | Male | | | 2.1 | 12.5 | Nucleus CI24RE(Ca) | ACE | 2 | Pre- |  |
|  | | | | | | | | | | | | |
