# Supplementary material for: Consonant and Vowel Confusions in Well-Performing Children and Adolescents With Cochlear Implants, Measured by a Nonsense Syllable Repetition Test
Source: Front Psychol. 2019 Aug 14;10:1813. doi: 10.3389/fpsyg.2019.01813 (PMC6702790; doi:10.3389/fpsyg.2019.01813)
Supplement: Supplementary file 2 [file Table_2.docx]

**Table S2 | Test results of participants with CIs**

| **Patient no.** | **HIST Monosyllable score (%)** | **NSRS (%)** | **NSRS-C (%)** | **NSRS-V (%)** |
| --- | --- | --- | --- | --- |
| 1 | 88 | 69.3 | 70.8 | 66.7 |
| 2 | 86 | 72.0 | 68.8 | 77.8 |
| 3 | 86 | 82.7 | 79.2 | 88.9 |
| 4 | 94 | 74.7 | 72.9 | 77.8 |
| 5 | 90 | 77.3 | 70.8 | 88.9 |
| 6 | 90 | 57.3 | 52.1 | 66.7 |
| 7 | 90 | 72.0 | 68.8 | 77.8 |
| 8 | 72 | 56.0 | 50.0 | 66.7 |
| 9 | 86 | 65.3 | 58.3 | 77.8 |
| 10 | 76 | 78.7 | 72.9 | 88.9 |
| 11 | 90 | 78.7 | 72.9 | 88.9 |
| 12 | 92 | 85.3 | 77.1 | 100.0 |
| 13 | 98 | 81.3 | 77.1 | 88.9 |
| 14 | 84 | 84.0 | 81.3 | 88.9 |
| 15 | 96 | 80.0 | 75.0 | 88.9 |
| 16 | 82 | 60.0 | 56.3 | 66.7 |
| 17 | 90 | 78.7 | 72.9 | 88.9 |
| 18 | 94 | 77.3 | 70.8 | 88.9 |
| 19 | 86 | 65.3 | 64.6 | 66.7 |
| 20 | 94 | 81.3 | 70.8 | 100.0 |
| 21 | 80 | 65.3 | 58.3 | 77.8 |
| 22 | 80 | 69.3 | 64.6 | 77.8 |
| 23 | 92 | 76.0 | 68.8 | 88.9 |
| 24 | 80 | 80.0 | 75.0 | 88.9 |
| 25 | 94 | 86.7 | 79.2 | 100.0 |
| 26 | 78 | 70.7 | 60.4 | 88.9 |
| 27 | 82 | 70.7 | 60.4 | 88.9 |
| 28 | 86 | 70.7 | 72.9 | 66.7 |
| 29 | 84 | 80.0 | 68.8 | 100.0 |
| 30 | 100 | 82.7 | 79.2 | 88.9 |
| 31 | 96 | 76.0 | 68.8 | 88.9 |
| 32 | 82 | 80.0 | 68.8 | 100.0 |
| 33 | 88 | 89.3 | 83.3 | 100.0 |
| 34 | 76 | 80.0 | 75.0 | 88.9 |
| 35 | 80 | 77.3 | 64.6 | 100.0 |
| 36 | 88 | 74.7 | 72.9 | 77.8 |
