# Supplementary material for: Consonant and Vowel Confusions in Well-Performing Children and Adolescents With Cochlear Implants, Measured by a Nonsense Syllable Repetition Test
Source: Front Psychol. 2019 Aug 14;10:1813. doi: 10.3389/fpsyg.2019.01813 (PMC6702790; doi:10.3389/fpsyg.2019.01813)
Supplement: Supplementary file 3 [file Table_3.docx]

**Table S3 | The VCV and CVC nonsense syllables included in the test**

| **Number°** | **aCa-syllables** | **iCi-syllables** | **uCu-syllables** | **bVb-syllables** |
| --- | --- | --- | --- | --- |
| 1 | ['ɑːbɑ] | ['iːbi] | ['uːbu] | [bɑːb] |
| 2 | ['ɑːdɑ] | ['iːdi] | ['uːdu] | [beːb] |
| 3 | ['ɑːfɑ] | ['iːfi] | ['uːfu] | [biːb] |
| 4 | ['ɑːɡɑ] | ['iːɡi] | ['uːɡu] | [buːb] |
| 5 | ['ɑːhɑ] | ['iːhi] | ['uːhu] | [bʉːb] |
| 6 | ['ɑːʝɑ] | ['iːʝi] | ['uːʝu] | [byːb] |
| 7 | ['ɑːkɑ] | ['iːki] | ['uːku] | [bæːb] |
| 8 | ['ɑːlɑ] | ['iːli] | ['uːlu] | [bøːb] |
| 9 | ['ɑːmɑ] | ['iːmi] | ['uːmu] | [bɔːb] |
| 10 | ['ɑːnɑ] | ['iːni] | ['uːnu] |  |
| 11 | ['ɑːpɑ] | ['iːpi] | ['uːpu] |  |
| 12 | ['ɑːsɑ] | ['iːsi] | ['uːsu] |  |
| 13 | ['ɑːtɑ] | ['iːti] | ['uːtu] |  |
| 14 | ['ɑːvɑ] | ['iːvi] | ['uːvu] |  |
| 15 | ['ɑːʃɑ] | ['iːʃi] | ['uːʃu] |  |
| 16 | ['ɑːŋɑ] | ['iːŋi] | ['uːŋu] |  |
| 17 | ['ɑːɾɑ] | ['iːɾi] | ['uːɾu] |  |
| 18 | ['ɑːçɑ] | ['iːçi] | ['uːçu] |  |
| 19 | ['ɑːɖɑ] | ['iːɖi] | ['uːɖu] |  |
| 20 | ['ɑːɽɑ] | ['iːɽi] | ['uːɽu] |  |
| 21 | ['ɑːɭɑ] | ['iːɭi] | ['uːɭu] |  |
| 22 | ['ɑːʈɑ] | ['iːʈi] | ['uːʈu] |  |
| 23 | ['ɑːɳɑ] | ['iːɳi] | ['uːɳu] |  |
| 24 | ['ɑːɲɑ] | ['iːɲi] | ['uːɲu] |  |
| 25 | ['ɑːʎɑ] | ['iːʎi] | ['uːʎu] |  |
| 26 | ['ɑːʁɑ] | ['iːʁi] | ['uːʁu] |  |
| 27 | ['ɑːrɑ] | ['iːri] | ['uːru] |  |
| **°** Numbers 1–16 contain speech sounds common in all Norwegian dialects, and were included in the analyses. | | | | |
