# Supplementary material for: Consonant and Vowel Confusions in Well-Performing Children and Adolescents With Cochlear Implants, Measured by a Nonsense Syllable Repetition Test
Source: Front Psychol. 2019 Aug 14;10:1813. doi: 10.3389/fpsyg.2019.01813 (PMC6702790; doi:10.3389/fpsyg.2019.01813)
Supplement: Supplementary file 4 [file Table_4.docx]

**Table S4 | Confusion matrix for NH 6-year-olds (N = 17); consonant repetitions in the aCa, iCi, and uCu contexts added together.**

|  |  |  | **Response** | | | | | | | | | | | | | | | | | | | | | | | | | | | | | | | |  | |  |  |
| --- | --- | --- | --- | --- | --- | --- | --- | --- | --- | --- | --- | --- | --- | --- | --- | --- | --- | --- | --- | --- | --- | --- | --- | --- | --- | --- | --- | --- | --- | --- | --- | --- | --- | --- | --- | --- | --- | --- |
|  |  |  | **Unvoiced** | | | | | | | |  | **Voiced** | | | | | | | | | | | | | | | | | | | | | | |  | |  |  |
|  |  |  | **S** | | |  | **F** | | | |  | **S** | | | | |  | | **F** | | | |  | | **Na** | | | | | |  | | **L** | |  | |  |  |
| **Stimulus** | | | **/p/** | **/t/** | **/k/** |  | **/s/** | **/ʃ/** | **/f/** | **/h/** |  | **/b/** | **/d/** | | **/ɡ/** | |  | | **/j/** | | **/v/** | |  | | **/n/** | | **/m/** | | **/ŋ/** | |  | | **/l/** | |  | | **U** | **Sum** |
| **Unvoiced** | **S** | **/p/** | 44 |  |  |  |  |  | 5 |  |  | 2 |  |  | |  | |  | |  | |  | |  | |  | |  | |  | |  | |  | |  | | 51 |
|  |  | **/t/** |  | 49 |  |  |  |  |  |  |  |  |  |  | |  | |  | |  | |  | |  | |  | |  | |  | |  | |  | | 2 | | 51 |
|  |  | **/k/** |  | 2 | 44 |  |  |  |  |  |  |  |  | 2 | |  | |  | |  | |  | |  | |  | |  | |  | |  | |  | | 3 | | 51 |
|  | **F** | **/s/** |  |  |  |  | 47 |  | 1 |  |  |  |  |  | |  | |  | |  | |  | |  | |  | |  | |  | |  | |  | | 3 | | 51 |
|  |  | **/ʃ/** |  |  |  |  | 2 | 47 |  |  |  |  |  |  | |  | |  | |  | |  | |  | |  | |  | |  | |  | |  | | 2 | | 51 |
|  |  | **/f/** | 1 |  |  |  |  |  | 49 |  |  |  |  |  | |  | |  | |  | |  | |  | |  | |  | |  | |  | |  | | 1 | | 51 |
|  |  | **/h/** |  |  |  |  |  |  | 1 | 47 |  |  |  |  | |  | | 1 | |  | |  | |  | |  | |  | |  | |  | |  | | 2 | | 51 |
| **Voiced** | **S** | **/b/** | 3 |  |  |  |  |  |  |  |  | 42 | 1 |  | |  | |  | | 5 | |  | |  | |  | |  | |  | |  | |  | |  | | 51 |
|  |  | **/d/** |  |  |  |  |  |  |  |  |  | 2 | 48 |  | |  | |  | |  | |  | |  | |  | |  | |  | |  | |  | | 1 | | 51 |
|  |  | **/ɡ/** |  |  | 1 |  |  |  |  |  |  |  |  | 48 | |  | |  | |  | |  | |  | |  | |  | |  | |  | |  | | 2 | | 51 |
|  | **F** | **/j/** | 1 |  |  |  |  |  |  | 2 |  |  |  |  | |  | | 44 | |  | |  | |  | |  | |  | |  | |  | |  | | 4 | | 51 |
|  |  | **/v/** |  |  |  |  | 1 |  | 1 |  |  | 1 |  |  | |  | |  | | 47 | |  | |  | |  | | 1 | |  | |  | |  | |  | | 51 |
|  | **N** | **/n/** |  |  |  |  |  |  |  |  |  |  | 1 |  | |  | |  | |  | |  | | 41 | | 1 | |  | |  | | 1 | |  | | 7 | | 51 |
|  |  | **/m/** |  |  |  |  |  |  |  |  |  | 2 |  |  | |  | |  | | 1 | |  | | 3 | | 42 | | 2 | |  | |  | |  | | 1 | | 51 |
|  |  | **/ŋ/** |  |  |  |  |  |  |  |  |  |  |  |  | |  | |  | | 1 | |  | | 2 | | 10 | | 32 | |  | |  | |  | | 6 | | 51 |
|  | **L** | **/l/** |  |  |  |  |  |  |  |  |  |  |  |  | |  | |  | |  | |  | |  | |  | |  | |  | | 38 | |  | | 13 | | 51 |
| S = stops; F = fricatives; Na = nasals; L = the lateral [l]; U = unclassified. | | | | | | | | | | | | | | | | | | | | | | | | | | | | | | | | | | | | | | |
