# Supplementary material for: Consonant and Vowel Confusions in Well-Performing Children and Adolescents With Cochlear Implants, Measured by a Nonsense Syllable Repetition Test
Source: Front Psychol. 2019 Aug 14;10:1813. doi: 10.3389/fpsyg.2019.01813 (PMC6702790; doi:10.3389/fpsyg.2019.01813)
Supplement: Supplementary file 6 [file Table_6.docx]

**Table S6 | Confusion matrix for NH 6-year-olds (N = 17); vowel repetitions in the bVb context**

|  | **Response** | | | | | | | | | |  |
| --- | --- | --- | --- | --- | --- | --- | --- | --- | --- | --- | --- |
| **Stimulus** | **/bɑːb/** | **/beːb/** | **/biːb/** | **/buːb/** | **/bʉːb/** | **/byːb/** | **/bæːb/** | **/bøːb/** | **/bɔːb/** | **U** | **Sum** |
| /bɑːb/ | 17 |  |  |  |  |  |  |  |  |  | 17 |
| /beːb/ |  | 15 | 1 |  |  |  | 1 |  |  |  | 17 |
| /biːb/ |  |  | 16 |  |  |  |  |  |  | 1 | 17 |
| /buːb/ |  |  |  | 17 |  |  |  |  |  |  | 17 |
| /bʉːb/ |  |  |  |  | 16 |  |  |  |  | 1 | 17 |
| /byːb/ |  |  | 4 |  | 2 | 11 |  |  |  |  | 17 |
| /bæːb/ | 2 |  |  |  |  |  | 14 |  |  | 1 | 17 |
| /bøːb/ |  |  |  |  | 3 |  |  | 14 |  |  | 17 |
| /bɔːb/ |  |  |  |  |  |  |  |  | 17 |  | 17 |
| U = unclassified. | | | | | | | | | | | |
