# Supplementary material for: Consonant and Vowel Confusions in Well-Performing Children and Adolescents With Cochlear Implants, Measured by a Nonsense Syllable Repetition Test
Source: Front Psychol. 2019 Aug 14;10:1813. doi: 10.3389/fpsyg.2019.01813 (PMC6702790; doi:10.3389/fpsyg.2019.01813)
Supplement: Supplementary file 7 [file Table_7.docx]

**Table S7 | Confusion matrix for NH 13-year-olds (N = 12); consonant repetitions in the aCa, iCi, and uCu contexts added together**

|  |  |  | **Response** | | | | | | | | | | | | | | | | | | | | |  |  |  |
| --- | --- | --- | --- | --- | --- | --- | --- | --- | --- | --- | --- | --- | --- | --- | --- | --- | --- | --- | --- | --- | --- | --- | --- | --- | --- | --- |
|  |  |  | **Unvoiced** | | | | | | | |  | **Voiced** | | | | | | | | | | | |  |  |  |
|  |  |  | **S** | | |  | **F** | | | |  | **S** | | |  | **F** | |  | **Na** | | |  | **L** |  |  |  |
| **Stimulus** | | | **/p/** | **/t/** | **/k/** |  | **/s/** | **/ʃ/** | **/f/** | **/h/** |  | **/b/** | **/d/** | **/ɡ/** |  | **/j/** | **/v/** |  | **/n/** | **/m/** | **/ŋ/** |  | **/l/** |  | **U** | **Sum** |
| **Unvoiced** | **S** | **/p/** | 36 |  |  |  |  |  |  |  |  |  |  |  |  |  |  |  |  |  |  |  |  |  |  | 36 |
|  |  | **/t/** |  | 35 |  |  | 1 |  |  |  |  |  |  |  |  |  |  |  |  |  |  |  |  |  |  | 36 |
|  |  | **/k/** |  |  | 36 |  |  |  |  |  |  |  |  |  |  |  |  |  |  |  |  |  |  |  |  | 36 |
|  | **F** | **/s/** |  |  |  |  | 33 |  |  |  |  |  |  |  |  |  |  |  |  |  |  |  |  |  | 3 | 36 |
|  |  | **/ʃ/** |  |  |  |  |  | 36 |  |  |  |  |  |  |  |  |  |  |  |  |  |  |  |  |  | 36 |
|  |  | **/f/** |  |  |  |  |  |  | 36 |  |  |  |  |  |  |  |  |  |  |  |  |  |  |  |  | 36 |
|  |  | **/h/** |  |  |  |  |  |  |  | 35 |  |  |  |  |  |  |  |  |  |  |  |  |  |  | 1 | 36 |
| **Voiced** | **S** | **/b/** |  |  |  |  |  |  |  |  |  | 35 |  |  |  |  |  |  |  |  |  |  |  |  | 1 | 36 |
|  |  | **/d/** |  |  |  |  |  |  |  |  |  |  | 35 |  |  |  |  |  |  |  |  |  |  |  | 1 | 36 |
|  |  | **/ɡ/** |  |  |  |  |  |  |  |  |  |  |  | 36 |  |  |  |  |  |  |  |  |  |  |  | 36 |
|  | **F** | **/j/** |  |  |  |  |  |  |  |  |  |  |  |  |  | 36 |  |  |  |  |  |  |  |  |  | 36 |
|  |  | **/v/** |  |  |  |  |  |  |  |  |  |  |  |  |  |  | 36 |  |  |  |  |  |  |  |  | 36 |
|  | **N** | **/n/** |  |  |  |  |  |  |  |  |  |  |  |  |  |  |  |  | 36 |  |  |  |  |  |  | 36 |
|  |  | **/m/** |  |  |  |  |  |  |  |  |  |  |  |  |  |  |  |  | 2 | 33 |  |  |  |  | 1 | 36 |
|  |  | **/ŋ/** |  |  |  |  |  |  |  |  |  |  |  |  |  |  |  |  | 1 | 7 | 25 |  |  |  | 3 | 36 |
|  | **L** | **/l/** |  |  |  |  |  |  |  |  |  |  |  |  |  |  |  |  |  |  |  |  | 34 |  | 2 | 36 |
| S = stops; F = fricatives; Na = nasals; L = the lateral [l]; U = unclassified. | | | | | | | | | | | | | | | | | | | | | | | | | | |
