# Supplementary material for: Consonant and Vowel Confusions in Well-Performing Children and Adolescents With Cochlear Implants, Measured by a Nonsense Syllable Repetition Test
Source: Front Psychol. 2019 Aug 14;10:1813. doi: 10.3389/fpsyg.2019.01813 (PMC6702790; doi:10.3389/fpsyg.2019.01813)
Supplement: Supplementary file 9 [file Table_9.docx]

**Table S9 | Confusion matrix for NH 13-year-olds (N = 12); vowels in the bVb context**

|  | **Response** | | | | | | | | | | | | | | | | | | | |  |
| --- | --- | --- | --- | --- | --- | --- | --- | --- | --- | --- | --- | --- | --- | --- | --- | --- | --- | --- | --- | --- | --- |
| **Stimulus** | **/bɑːb/** | | **/beːb/** | | **/biːb/** | | **/buːb/** | | **/bʉːb/** | | **/byːb/** | | **/bæːb/** | | **/bøːb/** | | **/bɔːb/** | | **U** | | **Sum** |
| **/bɑːb/** | | 12 | |  | |  | |  | |  | |  | |  | |  | |  | |  | 12 |
| **/beːb/** | |  | | 12 | |  | |  | |  | |  | |  | |  | |  | |  | 12 |
| **/biːb/** | |  | |  | | 11 | |  | |  | | 1 | |  | |  | |  | |  | 12 |
| **/buːb/** | |  | |  | |  | | 12 | |  | |  | |  | |  | |  | |  | 12 |
| **/bʉːb/** | |  | |  | |  | |  | | 12 | |  | |  | |  | |  | |  | 12 |
| **/byːb/** | |  | |  | | 3 | |  | |  | | 9 | |  | |  | |  | |  | 12 |
| **/bæːb/** | |  | |  | |  | |  | |  | |  | | 12 | |  | |  | |  | 12 |
| **/bøːb/** | |  | |  | |  | |  | | 1 | |  | |  | | 11 | |  | |  | 12 |
| **/bɔːb/** | |  | |  | |  | |  | |  | |  | |  | |  | | 12 | |  | 12 |
| U = unclassified. | | | | | | | | | | | | | | | | | | | | | |
