# Supplementary material for: Consonant and Vowel Confusions in Well-Performing Children and Adolescents With Cochlear Implants, Measured by a Nonsense Syllable Repetition Test
Source: Front Psychol. 2019 Aug 14;10:1813. doi: 10.3389/fpsyg.2019.01813 (PMC6702790; doi:10.3389/fpsyg.2019.01813)
Supplement: Supplementary file 10 [file Table_10.docx]

| **Table S10 \| Crosstab for the perception of speech features (*N* = 36).** | | | |
| --- | --- | --- | --- |
| **Speech Feature Contrast** | **Age at onset of deafness** | **Correct Repetitions** | **Incorrect Repetitions** |
| Voicing vs. nonvoicing | Prelingually deaf | 1,171 | 39 |
|  | Postlingually deaf | 289 | 6 |
| Nasality vs. nonnasality | Prelingually deaf | 1,202 | 8 |
|  | Postlingually deaf | 292 | 1 |
| Stops vs. fricatives | Prelingually deaf | 921 | 18 |
|  | Postlingually deaf | 114 | 4 |
