# Supplementary material for: Consonant and Vowel Confusions in Well-Performing Children and Adolescents With Cochlear Implants, Measured by a Nonsense Syllable Repetition Test
Source: Front Psychol. 2019 Aug 14;10:1813. doi: 10.3389/fpsyg.2019.01813 (PMC6702790; doi:10.3389/fpsyg.2019.01813)
Supplement: Supplementary file 11 [file Data_Sheet_1.docx]

**Supplementary text 1. Recording and preparation of the nonsense syllable repetition test.**

**2.1. Sound editing**

The test words were spoken by a Norwegian female speech therapist and recorded with a Zoom H4n hard disk recorder in an anechoic chamber. The recorded wav files were imported into the Praat computer program (Boersma and Weenink, 2018). The files were cut and split so they all were of equal length, ~1.0 s, and then imported into Adobe Audition CS6, Ver. 5.0, Build 708 (San José, CA, USA, 2012), for noise reduction and editing.

We decided not to normalize the sound files, as the energy level varies naturally between different speech sounds; for instance, [uː] contains less energy than [ɑː]. A normalizing procedure would have made some speech sounds unnaturally loud and some unnaturally soft. Instead, we used the “Match volume” command in Adobe Audition and adjusted the blocks of data of the recorded aCa, iCi, and uCu words to keep the volume at an even level between the blocks of speech sounds in the same vowel context.

**2.2. Equipment and test setup**

We used the Matlab computer program (Mathworks, Nantick MA, 2013) to randomize the nonsense syllables to be played in permuted sequential blocks consisting of 9 aCa syllables, 9 iCi syllables, 9 uCu syllables, and 9 bVb syllables. This randomization was to prevent the transcribers from learning the order of the nonsense syllables and thus being biased in their transcription. By dividing the nonsense syllables into blocks of 9 similar units, we aimed to make the test less repetitive and easier for the participants than a complete randomization would be.

We presented the nonsense syllables with the SpchUtil, ver. 5 computer program (Freed, 2004). This program enables each sound file to be called upon, and the sound level for each file can be individually adjusted. Because the sound level varies across each word, we measured the maximum value of the instantaneous sound pressure level, F_max_, of each word in the listening position. F_max_ was registered manually on a Norsonic 110 sound level meter (Tranby, Norway). We decided to keep 65 dB(A) in the listening position as a desired average sound pressure level, and we measured the F_max_ of the nonsense syllable sound files in 2 series. We found that the average F_max_ of all the sound files in both series was 60 dB(A). We then added 5 dB to all the nonsense syllables in SpchUtil, as we maintained the assumption that the mutual loudness differences between the recorded nonsense syllables was equal to their natural loudness differences.
